# Robotic-assisted thoracic duct ligation surgery guided by intralymphatic injection of indocyanine green

**DOI:** 10.1093/jscr/rjag709

**Published:** 2026-08-14

**Authors:** Oleg A Karpov, Thabbta Vianna, Sanjeet Singh Avtaar Singh, Tom Blankenstein, John McLenachan, Malcolm Will

**Affiliations:** Edinburgh Medical School, The University of Edinburgh, Edinburgh EH8 9AG, United Kingdom; Department of Cardiothoracic Surgery, Royal Infirmary of Edinburgh, Edinburgh EH16 4SA, United Kingdom; Department of Cardiothoracic Surgery, Royal Infirmary of Edinburgh, Edinburgh EH16 4SA, United Kingdom; Department of Radiology, Royal Infirmary of Edinburgh, Edinburgh EH16 4SA, United Kingdom; Department of Cardiothoracic Anaesthesia, Royal Infirmary of Edinburgh, Edinburgh EH16 4SA, United Kingdom; Department of Cardiothoracic Surgery, Royal Infirmary of Edinburgh, Edinburgh EH16 4SA, United Kingdom

**Keywords:** thoracic duct ligation, indocyanine green, thoracic surgery, interventional radiology, robotic surgery

## Abstract

Post-operative chyle leak is an uncommon but significant complication that can delay recovery and increase morbidity. Thoracic duct ligation is a recognized treatment option but may be technically challenging due to poor intraoperative visualization and numerous anatomical variations. This case report describes the use of indocyanine green (ICG) administered via intralymphatic injections, facilitating intraoperative guidance for precise thoracic duct localization and ligation in a complex patient who developed a chyle leak following a bilateral neck dissection. The Da Vinci Surgical System fluorescence imaging enabled real-time visualization of the thoracic duct, allowing for accurate clip ligation. ICG revealed significant anatomical variation of the thoracic duct, underscoring its value. Fluorescence guided lymphatic mapping reduced uncertainty and minimized the need for histological confirmation of lymphatic structures. Intraoperative ICG fluorescence represents a valuable adjunct for thoracic duct identification, particularly in anatomically complex cases, and may improve the success rate of surgical management of chylothorax.

## Introduction

Post-operative chyle leak is a recognized complication that can significantly impair recovery. It is associated with delayed wound healing, increased hospital stay, immunosuppression and an increased risk of infection/sepsis, contributing to higher morbidity and mortality [1]. Chyle leaks most commonly arise from inadvertent injury to the small lymphatic vessels or the thoracic duct itself during thoracic, cervical or oropharyngeal procedures [1]. In some cases, a chyle leak can develop after lymphadenectomies in oncologic patients, preventing wound closure, and increasing the risk of infection. Initial management is typically conservative, with aims to reduce lymphatic flow through dietary fat restriction, medium-chain triglyceride diets, and somatostatin analogues with ongoing chest drainage. If conservative management fails, surgical management may be required with either mass lymphatic ligation, pleurodesis, or direct thoracic duct ligation [2]. Thoracic duct ligation may prove challenging due to the small calibre of lymphatic vessels and considerable anatomical variations between patients [2]; the use of the intraoperative indocyanine green (ICG) (an amphiphilic dye) proposes to improve visualization of the duct, reducing iatrogenic injury. This case report describes the successful perioperative use of ICG dye to facilitate precise thoracic duct identification during robotic-assisted thoracic surgery in a patient with a post-operative chyle leak and significant anatomical variation.

## Case report

A 59-year-old male patient underwent a total laryngectomy and radical bilateral neck dissection for a T4N1M0 laryngeal squamous carcinoma, where he developed postoperative complications as the left cervical wound failed to heal due to a persistent chyle leak. Initially, he was managed conservatively with lipid-poor total parenteral nutrition (TPN) and a stoma bag over the wound. Despite these measures, the leak persisted, and subsequent chest radiography and computed tomography imaging demonstrated bilateral pleural effusions. Given the failure of conservative therapy, the multidisciplinary team decided to proceed with direct thoracic duct ligation.

Pre-operative assessment identified a recently formed laryngectomy stoma with a superadded infection, bilateral pleural effusions, and bibasal mucous plugging, where each was posing a risk to airway management and one-lung ventilation (OLV) tolerance. The left pleural effusion was drained pre-operatively via an intercostal chest drain, and suction bronchoscopy was performed to clear secretions and optimize OLV oxygenation. The 7.0 tracheostomy was upsized to an 8.0 Portex tracheostomy over a gum elastic bougie, through which an EZ-Blocker was inserted to achieve OLV, without a double-lumen endotracheal tube. Additionally, the patient's profound hypoalbuminaemia secondary to prolonged lipid-poor TPN diet, alongside underlying chronic emphysema with limited ventilatory reserve, made placement of anaesthetic lines particularly challenging.

**Figure 1:**
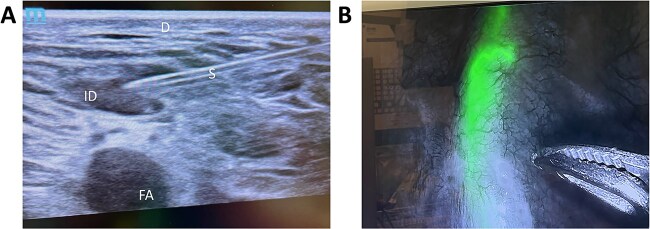
ICG dye injection and thoracic duct visualization. (A) Ultrasound-guided injection of ICG into the femoral lymphatic canal (inguinal lymphatic node). D: Dermis, S: Syringe, ID: Inguinal duct, FA: Femoral artery. (B) Fluorescent camera view from the Da Vinci robotic system illustrating the thoracic duct in stark green against the monochrome posterior mediastinum.

For intraoperative lymphatic mapping, 1–2 ml of ICG (at 2.5 mg/ml stock concentration) was administered bilaterally into inguinal lymph nodes under ultrasound guidance. This facilitated real time visualization of the thoracic duct and peritoneal lymphatics during the subsequent robotic-assisted thoracic procedure (Fig. 1A).

The patient was placed in the left lateral decubitus position, OLV was established, the Da Vinci system docked, and CO_2_ insufflation to 6 mmHg was used. Multiple intercostal blocks were performed with Levobupivacaine 0.25% and a paravertebral catheter was placed.

The thoracic cavity was inspected and an effusion of >500 ml was aspirated, during which marked anatomical variation was noted as the descending aorta was displaced laterally from the oesophagus. At this point, the near-infrared camera of the Da Vinci robot was utilized and the thoracic duct and associated lymphatic vessels were highlighted in green against surrounding structures (Fig. 1B). Further anatomical variation was noted as the thoracic duct was found overlying the descending aorta superficially. Unlike its usual left-sided course in close proximity to the oesophagus, the descending thoracic aorta in this patient was displaced to the right posterior mediastinum and separated from the oesophagus by several millimeters.

**Figure 2:**
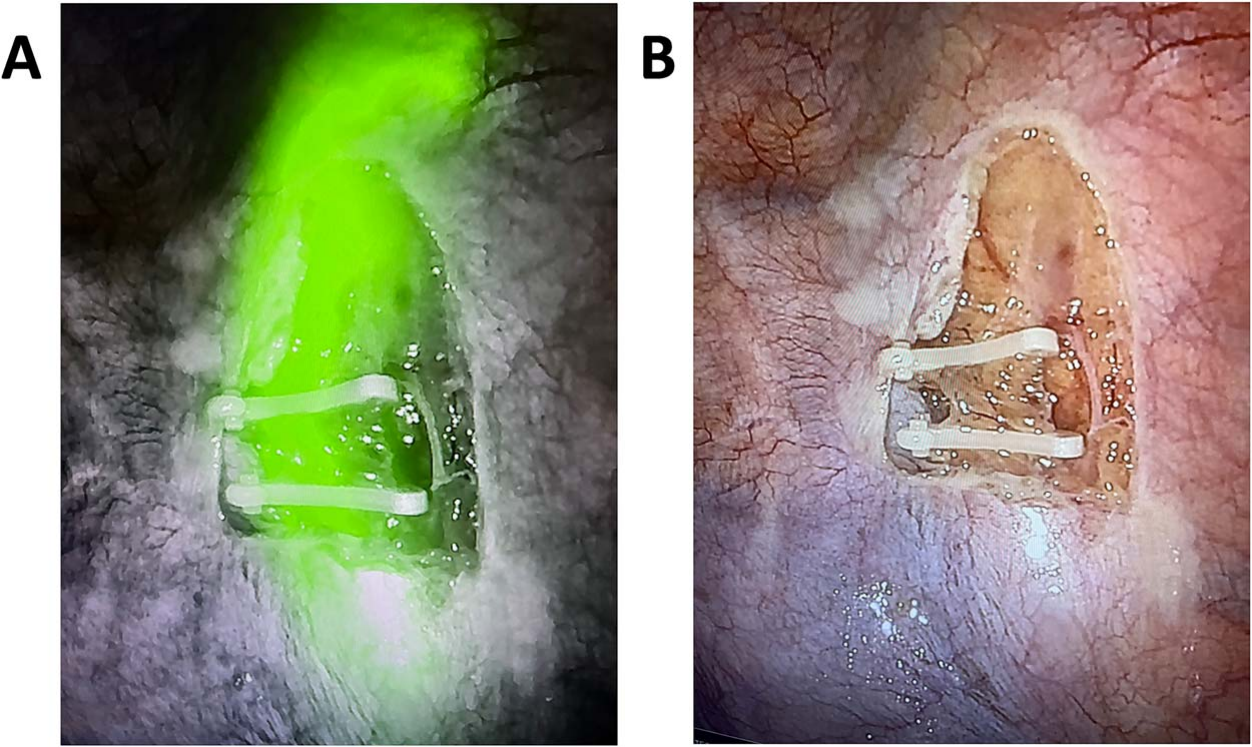
Thoracic duct clip ligation. Ligation outcome shown in near-infrared (A) and normal camera view (B).

The posterior mediastinal pleura was dissected, and the thoracic duct was isolated using a sling band. The thoracic duct was ligated bilaterally using Hem-o-lok clips to prevent chyle flow (Fig. 2). A chest drain was inserted and connected to a digital drainage system, ports were removed, the lung re-expanded, wounds were closed in layers, and the patient was weaned off anaesthesia for recovery in the Cardiothoracic ICU. Chyle leak ceased on postoperative day 3.

## Discussion

Postoperative chyle leak is a recognized complication that significantly increases morbidity and mortality, requiring early identification and timely management. Conservative management using a low-fat diet, somatostatin analogues, and chest drainage is initially preferred. Prolonged accumulation of chyle, particularly in high output leaks, is associated with a 25%–50% mortality rate due to severe malnutrition and immunosuppression [3]. If not improving, a surgical approach with a direct thoracic duct ligation is indicated [4]. Lymphatic flow is eventually restored due to collateral circulation following ligation.

In this case, the chyle leak originated from small lymphatic vessels distal to the thoracic duct. Normally these lymphatic channels carry chyle from lower extremities via cisterna chyli, located at L1-L2 adjacent to the abdominal aorta [3]. The thoracic duct (2-5 mm in diameter) arises from cisterna chyli and enters the thorax via the aortic hiatus, and ascends between the aorta and azygous vein [3]. The thoracic duct eventually empties into the venous circulation at the confluence of the left internal jugular and subclavian vein. Numerous tributaries, including the jugular and subclavian lymphatic trunks join the duct proximally, and these are at risk of injury during cervical dissection, necessitating thoracic duct ligation [5]. As many as 40%–60% of patients exhibit anatomical variations of the thoracic duct, contributing to the perioperative challenges of identification and ligation of the correct structure [6].

The use of perioperative lymphatic dyes offers a significant advantage in such complex scenarios. Near-infrared fluorescent light (700–900 mm) is particularly well suited to robotic-assisted surgery due to its high sensitivity, real time visualization, and excellent spatial resolution [7], and fluorescence-guided thoracoscopic surgery has demonstrated utility in chylothorax management [7, 8]. In this case, ICG fluorescence enabled clear delineation of the thoracic duct despite marked anatomical variation, facilitating accurate ligation. To our knowledge, this represents one of the first cases to utilize ICG to localize the thoracic duct for ligation following a total laryngectomy with bilateral neck dissection.
